# A qualitative study of the acceptability of remote electronic bednet use monitoring in Uganda

**DOI:** 10.1186/s12889-022-13393-5

**Published:** 2022-05-19

**Authors:** Sarah M. Alexander, Alfred Agaba, Jeffrey I. Campbell, Nuriat Nambogo, Carol S. Camlin, Mallory Johnson, Grant Dorsey, Kristian R. Olson, David R. Bangsberg, Ryan W. Carroll, Data Santorino, Paul J. Krezanoski

**Affiliations:** 1 Children’s National Hospital, 111 Michigan Ave NW, Washington, D.C, 20010 USA; 2 University of California San Francisco, San Francisco, CA USA; 3 Mbarara University of Science and Technology, Mbarara, Uganda; 4 Harvard Medical School, Boston, MA USA; 5 Boston Children’s Hospital, Boston, MA USA; 6 Consortium for Affordable Medical Technologies, Mbarara, Uganda; 7 Massachusetts General Hospital, Boston, MA USA; 8 Oregon Health and Science University-Portland State University School of Public Health, Portland, OR USA; 9 Opportunity Solutions International, San Francisco, CA USA

**Keywords:** Malaria, Bednets, LLIN, Prevention, Adherence monitoring, Acceptability

## Abstract

**Background:**

Distribution of long-lasting insecticide treated nets (LLINs) is the most widely used intervention for the prevention of malaria but recall and social desirability biases may lead to challenges in accurately measuring use of bednets. SmartNet is a remote electronic monitor that provides objective measurements of bednet use over weeks at a time. Assessing local acceptability is important when implementing innovative global health technologies such as SmartNet. This study draws on established models such as the Technology Acceptance Model (TAM) and Theoretical Framework of Acceptability (TFA) to assess acceptability of SmartNet in Ugandan households.

**Methods:**

Semi-structured qualitative interviews were conducted at weeks one and six following installation of SmartNet in ten households in Western Uganda. Heads-of-households answered open-ended questions addressing the main acceptability domains of the TFA and TAM models (i.e. perceived ease of use, ethicality, etc.). Responses were digitally recorded, transcribed, coded and analyzed using a thematic analysis approach.

**Results:**

Seven out of ten households interviewed reported no difference in use between SmartNet and a standard LLIN. Households stated the large size, soft fabric, and the efficacy of SmartNet relative to a standard LLIN contributed to perceived usefulness and perceived ease of use. Opportunity costs of the novel monitoring system expressed by households included difficulty washing nets and dislike of blinking lights on the device. Barriers to SmartNet use focused on questions of the ethics of bednet use monitoring, discomfort with technical aspects of the device and a poor understanding of its function amongst others in the community. However, explaining SmartNet to other community members resolved these concerns and often resulted in interest and acceptance among peers.

**Conclusion:**

Objective monitoring of bednet use with SmartNet appears acceptable to these households in Uganda. Use of SmartNet seems to be similar to behaviors around use of standard LLINs. Viewpoints on many aspects of SmartNet were generally favorable. Concerns around ethicality of bednet monitoring are present and indicate the need for continuing community education. The device will continue to be optimized to make it more acceptable to users and to accurately reflect standard LLIN use to improve our understanding of prevention behaviors in malaria endemic settings.

**Supplementary Information:**

The online version contains supplementary material available at 10.1186/s12889-022-13393-5.

## Background

Malaria killed an estimated 409,000 people worldwide in 2019 and is a major health burden in sub-Saharan African countries like Uganda [1]. Long-lasting insecticide-treated bednets (LLINs) are the most common and effective means of individual protection from malaria [2]. LLINs are widely available via free universal distribution campaigns and the World Health Organization (WHO) recommends that every individual at risk of malaria, over 3 billion people worldwide [3], sleep under an LLIN each night.

Given increasing access to LLINs, it is important to assess how these bednets are used under “real world” conditions. Due to the challenge of social desirability and recall bias in using self-reports as a measure of bednet use [4], new approaches have sought to obtain more objective measures of individual and household use behaviors [5, 6]. One such tool is SmartNet, a remote electronic bednet monitor that provides objective measurements of bednet use over weeks at a time. SmartNet is designed to be used as a typical LLIN with the capacity to measure bednet use. We have published previously on attitudes about SmartNet and the concept of objective bednet use monitoring among mothers in Uganda [7]. In addition, we have published pilot data demonstrating the feasibility of SmartNet to capture patterns of bednet use among ten households in western Uganda [8]. We now extend this work to understand acceptability of the SmartNet device in the receiving population.

Best practices in the life cycle of developing innovative global health technologies call for the assessment of local acceptability of novel technologies [9]. Failure to assess acceptability of proposed tools limits use among target populations and is one of the major barriers in transitioning from prototype to widespread use [10, 11]. The Technology Acceptance Model (TAM) is used to assess acceptability of new technologies. The TAM posits that “Perceived Ease of Use” and “Perceived Usefulness” are the main contributors to the formation of “Attitude” and the eventual “Behavior Intention” to use, which is crucial to acceptance of the technology [12]. The theoretical framework of acceptability (TFA) is another model that addresses elements specific to global health contexts, such as ethical consequences and opportunity costs of using the technology, which may vary based on local cultural norms [13].

This study aimed to evaluate the acceptability of the SmartNet objective bednet monitoring tool, and of objective bednet monitoring more broadly. This was done by applying the TAM and TFA acceptability models using semi-structured qualitative interviews with ten households in Western Uganda actively using SmartNet for 6 weeks each. Opinions were solicited at multiple time points during the study to evaluate for changes in attitudes after using the SmartNet. The goal of this study was to explore and understand acceptability, and possible improvements, for future use of SmartNet in research settings. Additionally, it aimed to provide insight into factors that enhance (or impede) acceptability of bednet use monitoring more generally to inform the development of new approaches for assessing LLIN adherence in households at risk of malaria.

## Methods

### Study setting

As previously described, ten households were recruited from the Kinoni Health Centre IV in the Mbarara district of southwestern Uganda [8]. Details about the study site have been published elsewhere, but in brief, the households that participated in this study were derived from an area in Southwestern Uganda with moderate holoendemic malaria transmission [8]. The most recent universal LLIN distribution campaign was conducted in the area in 2013–2014.

### Participant selection

Invitations to participate were extended to women presenting to the clinic who had at least one child under 5 years of age or who were pregnant. Recruitment was done by convenience sampling. Women who presented for pre-natal visits were identified by the nurses and then introduced to a research assistant to discuss possible enrollment. Written consent was obtained and no participants identified as illiterate. Once women chose to participate, their homes were visited by study personnel for the consent process and SmartNet installation. The recruited mothers provdied written consent on behalf of the household for the installation and study procedures. The qualitative interviews were performed within a pilot study of the use of SmartNet in 10 households. The sample size for the qualitative interviews was thus determined by the total number of households using SmartNet and thus who had long term experience using SmartNet. Previous studies have shown thematic saturation can be achieved in as few as 6–10 interviews, so a sample size of 10 was not felt to be limiting in this study [14, 15].

### Study design

One SmartNet was installed over a commonly used sleeping area per household preference. Study personnel demonstrated how SmartNet is hung and used like a standard LLIN, but no further education was given in order to minimize the distinctions between SmartNet and a standard LLIN. Participants were instructed to use the SmartNet the same way they would use a standard LLIN. Demographic data about the households were also collected at this time through a structured survey questionnaire. SmartNets are comprised of conductive material interwoven into a WHO-approved LLIN and a small attached electronic receiver, with a light that blinks periodically every 15 minutes to ensure it is on and collecting data (see reference 8 for photos). After 1 week, an initial semi-structured acceptability interview was conducted by a trained qualitative interviewer with the recruited mother of the household. One research assistant (co-author AA) performed the interviews, and he was fluent in both English and Runyankole. Interviews consisted of 8 open ended questions, discussed in English or Runyankole as preferred by the participant, with interview length determined by the time participants needed to adequately answer the questions (Interview Guide available in Appendix A). Study personnel continued to visit the household weekly for the first 4 weeks to collect SmartNet electronic data and perform technical checks. After 6 weeks, a final semi-structured acceptability interview was performed using the same interview guide, with the same head of household member, the SmartNet was removed, and households were provided with a new, standard LLIN.

### Data collection and analysis

The COREQ checklist was followed in reporting and analyzing the following qualitative data [16]. The ten participant head of households answered the same open-ended questions at weeks one and six about their impressions of SmartNet, individual concerns, perceived differences between a standard LLIN and SmartNet, any change in LLIN use since using the SmartNet, recommendations for potential improvements and any concerns about the device from the viewpoint of others outside of their household. The responses were recorded digitally and later transcribed and translated from the digital audio recordings.

We used a thematic analysis approach to analyze transcribed interviews [17]. First, all interviews were reviewed and structured and inductive codes were created. Structured codes were adapted from the interview guide and specifically identified responses related to 1) who should use a bednet and 2) differences between SmartNet and LLIN use. Inductive codes were developed to identify unanticipated concepts that emerged from interviews. As review of transcripts progressed, structured and inductive codes were organized in a codebook. The transcripts were coded independently and without the use of software by two members of the research team (SMA and PJK). Researchers discussed inconsistencies in coding, and when inconsistencies existed, a final code was established by consensus between the coders.

Coded text was then reviewed by the research team and categorized into themes. We used a hybrid approach to developing themes. Because we sought to understand acceptability using concepts from TAM and TFA, deductive themes representing key constructs from these theories were developed. Deductive themes were also developed to reflect similarities/differences between SmartNet and LLIN use. Meanwhile, inductive themes were elaborated to reflect concepts that emerged in our analysis. This hybrid analytic strategy enabled us to understand key issues related to acceptability of a novel technology (as informed by prior technology acceptability theories), while also exploring culture- and context-specific concepts related to technology acceptability not fully captured in existing theory. We used simple count data to describe frequency of key themes.

In this study, credibility was established through the longitudinal design of the study; iterative codebook development and parallel coding by two researchers extensively familiar with the research environment and SmartNet design; repeat immersion/crystallization analysis whereby we repeatedly returned to interview transcripts as themes were developed; and peer debriefing to enable external evaluation of deductive and inductive themes.

## Results

### Demographic data

Detailed demographic data has been published elsewhere, but overall, the 10 households consisted of 1.3 (SD: 0.5; range 1–2) children younger than 5 years of age and a total of 4.9 members each (SD: 1.8; range: 3–8) [8]. Four out of the 10 households included one pregnant woman. Households possessed on average 2.5 bednets per household (SD: 0.8), leading to a ratio of occupants to bednets of 2.0 (SD: 0.3). Eight of the mothers had a primary school education, and two had a university education. Main income activities were diverse and included farming, trade and butchery. SmartNet was used nightly in six households by one adult and one child younger than 5 years old. In two households, the SmartNet was used only by adults, and in the remaining two households only by children. Most households (88%) had received their LLINs from free distribution campaigns.

### Themes

Themes are identified below with supporting quotations. Of note, the TFA has several constructs, but through the inductive coding process only the constructs of Opportunity Costs and Ethicality arose in interviews and are discussed. A summary of the thematic breakdown can be viewed in Table 1.

**Table 1:** Major and Minor Themes identified in interviews with SmartNet users

**Who should use LLINs**	
Children, pregnant women, and other high-risk individuals should use LLIN	
Everyone should use LLIN but prioritize high risk groups	
**LLIN compared to SmartNet**	
**Differences between using LLIN and SmartNet**	
No difference	
Improved use due sense of accountability and monitoring	
Improved use due to education about net use	
**Perceived differences between characteristics of LLIN and SmartNet**	
No difference	
SmartNet is larger and more effective than prior LLIN	
SmartNet light may be bothersome	
SmartNet light may be reassuring	
**TAM and TFA constructs**	
**Perceived ease of use of SmartNet**	
SmartNet is high-quality, ergonomic, and durable	
Education about SmartNet facilitates net use	
**Perceived usefulness of SmartNet**	
SmartNet works better and prevents mosquitos from entering more effectively than LLIN	
SmartNet decreases frequency of malaria	
SmartNet use made participants feel “cared for”	
**Opportunity costs of using SmartNet compared to LLIN**	
Difficulty washing SmartNet	
SmartNet size not always sufficient	
Insecticide treatment will wear off, and SmartNet might not be retreated	
**Ethical considerations**	
Concerns about “spying” in participants homes	
Accountability provides positive motivation to use the net	
Fear of judgement for not using LLIN	
Concerns about SmartNet may be linked to users’ understanding	
**Recommended improvements**	
Remove SmartNet’s blinking light	
Provide a storage bag to protect electronic components of SmartNet	
Community education	
Increase size and attachment options	
Provide insecticide re-treatment for SmartNet	

### Who should use LLINs

Head of households were asked about who they thought should use bednets. All respondents reported the belief that pregnant women and children, especially 5 years old and younger, should use LLINs to prevent malaria. Certain participants widened the criteria to those with chronic illness or those perceived to have a vulnerability to malaria. Seven of the ten households expressed that everyone should use an LLIN, but when faced with limited resources, LLINs should go first to young children and pregnant women.

“Personally I think it should be used by … children who are the age of 5 years and below and also pregnant mothers.” (Household 1, woman 35 years)

“It should be used by a pregnant mother or a child below the age of 5 and even the adults because we are all prone to catching malaria but the group we look at more is the pregnant mothers and children.” (Household 6, woman 28 years)

### Difference between use behaviors with standard LLIN versus SmartNet

Households were asked if their LLIN use behaviors changed with use of SmartNet. Six households reported no difference. One household noted an increase in their SmartNet use compared to standard LLIN use at the first week interview, but at the sixth week interview the interviewee no longer felt there was an increase.

“It has not changed anything because when you educated us, we understood and we know it helps to prevent malaria like the other usual net.” (Household 1, woman 35 years)

“No, it has not changed anything. It is also like any other nets.” (Household 3, woman 42 years)

The remaining households reported that SmartNet helped them improve their LLIN use through various means, including an improved understanding on how to use and hang the net, the SmartNet apparatus serving as a physical reminder to use the net, and serving as a means of accountability due to SmartNet’s recording function.

“Definitely it has changed something because when you taught me I gained more knowledge on how to use mosquito nets. Other times I would just use them any how in the wrong way but now I use them the proper way and I like using them.” (Household 8, woman 25 years)

“Every time you look at this device you are reminded that you need to put the net but you see the other usual net whether you put it there or not you know that there is nothing that will record you that you have used it.” (Household 6, woman 28 years)

### Perceptions of differences between LLINs and SmartNet

Some households noted differences between their previous LLIN and SmartNet. These differences included different size, fabric and perceived improved efficacy of the SmartNet. Some households felt the SmartNet size was larger than previous LLINs and they preferred SmartNet, while another household wished SmartNet could be even bigger.

“This one, the SmartNet might trap mosquitoes better than the other usual net. And you see for it, it covers the big bed while the other one only covers the small bed.” (Household 1, woman 35 years)

The main difference discussed was the SmartNet recording apparatus, which included a small box of wires and a blinking light. Some households liked the blinking light because they felt reassured the SmartNet was working, whereas other households felt it was bothersome.

“Yes, there has been some change. Mosquitoes can’t get inside [this] net and also the flash from its light easily reminds you that you are under a mosquito net.” (Household 2, woman 37 years)

“If there wasn’t the light it would be good because there are some people who have a problem with light at night when sleeping.” (Household 4, woman 31 years)

Notably, some households reported that there was minimal difference between SmartNet and their previous LLIN.

“There is no big difference only that this one records how you have been using the net. That is only difference I see.” (Household 5, woman 24 years)

### Perceived ease of use of SmartNet

According to the TAM model, perceived ease of use is an important aspect of the acceptability of new technologies. Several elements about the SmartNet device were identified as contributing to its ease of use, including the materials and size of the net. Although these features are not inherent to the recording technology, they are part of the SmartNet design which involved integration of the monitoring capability into high quality and locally available LLINs. The material’s texture was noted to be softer than previous LLINs, such that it did not irritate the skin. The material was also perceived to be folded easily without damage. Households remarked on a lack of insecticide smell and no overheating, both common issues with standard LLINs. SmartNet was noted by households to be the same size or larger than previous LLINs, allowing it to cover the entire sleeping area.

“This mosquito net is nice because when you put it on the bed and fold it on all corners of the bed it fits well and even if you sleep under it, it remains neat and it is not broken or damaged in any way … I liked it and in fact if you like you can continue for a whole year.” (Household 8, woman 25 years)

“The previous net … sometimes when your skin touched it you would feel itch and start scratching yourself. But I see this one have no problem.” (Household 4, woman 31 years)

“Because sometimes it [standard LLIN] has insecticide in it and when you sleep in it it makes you sneeze and uncomfortable which may cause you at times not to sleep in it every day. Other times you may find the net is hard and rough … But this net, the smart net I found it soft and when you fold it, it remains there and there is a way its design has impressed us.” (Household 3, woman 42 years)

Another component of usability expressed by the households was an increase in understanding about how to use LLINs that was facilitated by researchers explaining the SmartNet and how to hang it.

“Because you came and showed me how to use it and now I hang it neatly [and] well on the bed. Previously I would not mind how I put the net on the bed.” (Household 8, woman 25 years)

### Perceived usefulness of SmartNet compared to standard LLINs

The TAM model also identifies the perceived effectiveness of new technologies as an important domain relating to acceptability. Several households felt the mesh fabric and insecticide treatment were more effective than their previous LLINs. In particular, households referred to the mesh material as woven differently with smaller holes that didn’t allow mosquitos to penetrate.

“This net that you brought us is good because it is treated and mosquito can’t penetrate it while the other usual net allows mosquitoes to get inside.” (Household 10, woman 36 years)

“The change I saw is that this one [standard LLIN] has big holes while the one you gave me [SmartNet] has small holes, mosquitoes can’t go through them.” (Household 2, woman 37 years)

Some also commented that there was decreased incidence of malaria in their household during the period of SmartNet use.

“What I have noticed is that my child has been getting malaria yet she sleeps in a mosquito net but since she started sleeping under the smart net that has changed.” (Household 4, woman 31 years)

Households also spoke about the overall appeal of SmartNet to others in their community, and that many in their community expressed interest in using SmartNet. Households reported that they felt cared for by the SmartNet researchers and that there was an inherent usefulness of SmartNet to contribute to understanding of malaria.

“[K] nowing that our doctors care about us enough to study about why we sleep in mosquito nets but still get malaria makes me also to care enough and make sure that I sleep under a mosquito net.” (Household 3, woman 42 years)

Features of the SmartNet technology such as the visible wires and light also made some households feel the net was more useful than a standard LLIN, and the technology made it more appealing.

“They have liked this net [SmartNet] because when they saw these wires they thought that it works better than the other net [LLIN].” (Household 1, woman 35 years)

### Opportunity cost of using SmartNet compared to standard LLINs

The TFA model identifies perceived opportunity costs of using new technologies as a component of its acceptability. Objective bednet monitoring tools such as SmartNet come with inevitable opportunity costs of use. Perception of invasions of privacy is an important issue which is addressed more in depth in the next section on ethics of bednet monitoring. However, households expressed other concerns about the opportunity costs as well, identifying barriers inherent in the SmartNet technology such as difficulty washing nets with the recording device attached.

“Okay the modification I see that should be made is to create a side pocket with a zipper on the side of the net for this device … When you are going to wash the net you can remove the device put it somewhere and put it back in the bag when the net is dry.” (Household 6, woman 28 years)

In addition, one household remarked on limitations of size which prevented the net from covering the entire sleeping area.

“In order to make it better you should increase its size and make it bigger because some of our beds are big and it doesn’t fit well on them.” (Household 2, woman 37 years)

There was also worry that the insecticide treatment may wear off the nets over time, leading to decreased efficacy, and that the electrical components may impede retreatment with insecticide.

“If you think that the treatment in it doesn’t last long say it goes for only six months then you should always plan to have them treated again after that period.” (Household 9, woman 38 years)

### Ethics of objective bednet monitoring

Ethical considerations are another component of the TFA model that is particularly relevant to global health technologies used in diverse cultural settings. Undoubtedly, the process of objective bednet monitoring raises privacy and ethical concerns, both for research subjects and potential bystanders [18]. Households were asked about their own concerns related to bednet use monitoring. In addition, to avoid direct confrontation, households were also asked about their experience with, or perceptions of, concerns among those outside their household. Some common themes that arose included outsider fear of the technology and poor understanding of its functioning.

“Some of the people who have seen it when they see the other blinking light they think that you could be spying on us and our homes … but after sometime now people have come to understand that it’s all about research because of malaria.” (Household 3, woman 42 years)

Still others spoke positively about the accountability the monitoring provided.

“It helps to remind me when to put the net because it records what you are doing.” (Household 6, woman 28 years)

Several households noted other community members’ fear of behaviors being recorded while people are in bed. In addition, there was also a fear of judgment for recording poor LLIN use.

“They might not know how to use them or are not sleeping under them and that is why they don’t want people to know.” (Household 8, woman 25 years)

“For them they were scared of it thinking that it might be having video coverage capturing what you are doing. So when I explained that it wasn’t the case they liked it.” (Household 6, woman 28 years)

Some households also noted poor understanding of malaria and bednets in the community as a component affecting perceptions of the trade-off between the value of SmartNet monitoring and the intrusions necessary to gather the data.

“I think it depends on the understanding of the individual. After understanding that it protects against mosquitoes, I think they wouldn’t mind [being monitored].” (Household 1, woman 35 years)

All households shared that once they understood SmartNet they were able to use it comfortably and without fear. Notably, households shared that explaining SmartNet to others frequently resulted in interest amongst their peers.

“All of them have liked it and want to have it. Even some people come and when they see it they ask how they could get them and I tell them that they can’t easily get them now but they really liked it.” (Household 9, woman 38 years)

“What is important is to educate people about its importance. If people are aware about that they would have no problem with it.” (Household 3, woman 42 years)

### Potential improvements for SmartNet

Households shared multiple potential improvements for SmartNet that touched on the domains discussed above. These included minimizing the disagreeable appearance of the recording aspects of the device, including enclosing the device wires and covering the blinking light.

“Change that blinking device and maybe put it somewhere else because it scares people because you see other mosquito nets don’t have it. If it is not there I think people would appreciate the net more.” (Household 3, woman 42 years)

The light was stated to be bothersome to sleep, and also initially increased fears about the nature of the recording. Suggestions for improving the negative aspects of the appearance of the wires and light included placing it under the bed or adding a bag for the device to be hidden in. In addition, as mentioned above, an attached bag may allow the recording apparatus to remain protected when the net was being washed.

“Maybe create a bag for that device inside the net and put the device in the bag such that the child doesn’t see it and destroy it that would be better.” (Household 6, woman 28 years)

Households suggested broader community education around SmartNet.

“If we organize a workshop and educate them about its use and importance they would like it because when I was also taught about its importance I liked it.” (Household 3, woman 42 years)

Other recommendations addressed ease of use, including increasing the size of the net itself and providing hooks to improve the convenience of hanging the net. Other feedback related to effectiveness and maintenance, suggesting that insecticide treatment be reapplied on the net once it started to fade.

## Discussion

There is growing interest in the use of new remote and objective tools for measuring bednet use in households at risk of malaria. Acceptability of novel technologies in target populations is an important component of effectiveness. While initial pilot studies addressed attitudes about the SmartNet technology and the feasibility of measuring patterns of bednet use, this qualitative study explored domains related to SmartNet’s acceptability among users in Ugandan households. Comments from households in this study indicate a positive response towards accepting objective bednet monitoring, with addressable areas of improvement for aspects of the SmartNet technology.

The core tenets of acceptability according to TAM are perceived ease of use and perceived usefulness. Responses from households in this study revealed members’ thoughts within these domains and culminated in favorable attitudes about SmartNet. Thoughts about SmartNet fell into two main categories: features reflecting favorable impressions of characteristics of the LLINs used to produce the SmartNet (e.g. net size) and features inherent to the technology of SmartNet. SmartNet was deemed easy to use, chiefly because of its similarity to a standard LLIN and its high-quality materials. Common themes included satisfaction with the size and softness of the bednet, and materials that were foldable and durable. Households found value in utilizing SmartNet to help contribute to malaria research and perceived the visual display of electronic components on the device useful to improve their bednet adherence. A potential intervention to increase SmartNet use in future studies could be to provide the option for users to obtain an individualized report on their bednet adherence, helping them improve their bednet use. However, it is important to note that this approach would be at odds with the primary goal of SmartNet as discussed below, to obtain an understanding of use of bednets under real-world conditions.

Households also had high regard for the perceived usefulness of the net. They appreciated the mesh fabric and insecticide treatment’s ability to prevent the entry of mosquitos. All households indicated that the SmartNet was as good or better at preventing mosquito bites compared to their previous LLINs. It is important to note that SmartNet was modeled on a standard locally available LLIN in terms of size, shape, mesh hole size and insecticide treatment. The prevalence of comments about these aspects may indicate the households’ previous LLINs were damaged or made from lower quality materials.

Following well-established co-creation principles, SmartNet was designed with the involvement of local stakeholders at the Consortium for Affordable Medical Technologies office in Mbarara, Uganda [9]. Key components of SmartNet were intentionally manufactured in Uganda by employing a local seamstress to sew conductive fabric components into the standard LLINs using high quality local materials. Positive responses to the appearance and material of the net are likely important factors behind the overall positive response to SmartNet. We believe that early and continuous involvement of local stakeholders in the design and manufacturing of the device are key factors underlying the high acceptability of the technology.

Although many features of SmartNet were viewed positively, there were still notable opportunity costs and barriers to convenient use identified by the users. These included an inability to wash the nets, wrong sized nets, and concerns about long-term efficacy of the insecticide treatment. These concerns mirror common barriers to standard LLIN use that have arisen in other qualitative studies investigating general bednet use [19]. In future work, efforts will be made to minimize the barriers that are specific to the underlying technology in SmartNet such as adding a waterproof device covering to render the nets washable. The goal is to achieve a balance between acceptability, functionality, and, specifically for adherence monitoring tools like SmartNet, minimizing interference with typical behaviors.

An additional important barrier to the acceptability of a tool like SmartNet relates to the ethics of bednet monitoring. Although SmartNet does not collect audio or visual data, the idea of tracking night time behaviors creates an understandable privacy concern. These privacy concerns are best evaluated in comparison to other studies that use objective measurements of bednet use to overcome social desirability bias with, for example, unannounced night-time visits to determine who is using the bednet during sleeping hours [20–22]. While SmartNet presents certain privacy concerns, there is arguably a less dramatic invasion of privacy than with unannounced visits. Many households spoke about an initial fear of the device, typically reported as concerns relayed by a third party (i.e. others in the community), especially fear of monitoring behaviors in bed. In Uganda in particular, sexual behaviors can carry potential stigma which is important to address [14]. These concerns were exacerbated by a lack of understanding of specific elements of the SmartNet device, such as a blinking light on the electronic components. Redesigning elements of SmartNet, such as removing blinking lights, may help reduce fears of being recorded. Most importantly, explanations of the nature of the monitoring are critical for community acceptance. Households spoke about how explaining the device to their friends and family greatly improved attitudes towards SmartNet in their community, which shows promise for the acceptability of objective bednet monitoring.

The goal of any objective bednet measuring tool is to simulate the experience of typical use of LLINs while also gathering scientific data. In addition to the acceptability of using the tool, it is crucial to address whether the bednet behaviors measured with SmartNet reflect typical use of a standard LLIN. The main critique of subjective reporting of LLIN use is the predisposition towards recall and social desirability biases, and it is important to identify the degree to which such biases are avoided with objective measuring tools such as SmartNet. The results of this study suggest that while users are aware their bednet use is being recorded by SmartNet, SmartNet nevertheless achieves a measure of typical use behaviors. At the six-week mark, seven out of ten households reported no change in behavior from their normal LLIN use. Of the three households that reported deviation from normal LLIN use, they remarked on increased net use due to the accountability that the net offered them, as well as a visual reminder to use the net. Households also noted potential fears of judgment for not using their nets. These two themes, effect of accountability and fear of judgment, are similar to those most operative in social desirability bias seen with self-reporting. One household initially reported that the SmartNet led to an overall increase in net use, but by the six-week mark this household reported that they used SmartNet similarly to their standard LLIN. This could suggest that the accountability effect of objective measuring tools diminishes over time, resulting in a more representative documentation of actual behavior. While the awareness of being monitored is an unavoidable byproduct of the device, it is reassuring that for most participants (seven of ten households), reported SmartNet use correlated to their typical LLIN use. Using the SmartNet as an objective surveillance tool requires a continual emphasis that participant try to use the net as “typically” as possible, perhaps by continuing to reinforce that there is no penalty for not using the net.

This study contains certain limitations. Opinions were solicited from ten households using the SmartNet during a limited six-week period. In future studies, exploring attitudes from more households with longer use of SmartNet may yield a more diverse set of thoughts and experiences. Answers were primarily shared by the mother in the household, without providing a broader range of perspectives from men and children who may have differing views. Interviewing men and children for their attitudes on SmartNet will also be an area of future work. It is important to note that self-reported changes in bednet use and experiences of use are still subject to social desirability bias, although participants were aware their answers did not affect their access to SmartNet.

## Conclusion

Objective monitoring of bednet use via innovative tools such as SmartNet is acceptable to households in Uganda, and the devices appear to be used in a manner like typical LLINs. Explanations of the intent and function of SmartNet helped assuage individual and community concerns about nighttime monitoring. Future work will include improvements to the device to increase both SmartNet’s acceptability to users and its ability to accurately reflect typical LLIN use in malaria endemic settings.

## Supplementary Information

**Additional file 1: Appendix A.**

## Data Availability

The datasets used and/or analyzed during the current study are available from the corresponding author on reasonable request.

## Abbreviations

LLIN: Long-lasting insecticide treated nets
TAM: Technology Acceptance Model
TFA: Theoretical Framework of Acceptability
WHO: World Health Organization
