# Supplementary material for: A qualitative study of the acceptability of remote electronic bednet use monitoring in Uganda
Source: BMC Public Health. 2022 May 19;22:1010. doi: 10.1186/s12889-022-13393-5 (PMC9118600; doi:10.1186/s12889-022-13393-5)
Supplement: Supplementary file 1 — Additional file 1: Appendix A. [file 12889_2022_13393_MOESM1_ESM.docx]

**Appendix A.** Interview Guide

“Now I would like to have a conversation with you about SmartNet. I am going to use an audio recorder so that we can record this information and review it later to learn about what people here think about SmartNet.”

If you have used a bednet before, tell me about any differences you found between using a bednet and using the SmartNet.

Tell me about how using SmartNet changed how you or your family used bednets.

What did other people think about SmartNet if they saw it?

Are there ways that we can improve how SmartNet works?

Who should use SmartNets?

Did it bother you that your use of your bednet was being measured by the SmartNet?

Why do you think some people do not want their bednet use measured?

Are there ways that we can improve how SmartNet looks?
